# Kolliker’s Organ and Its Functional Role in the Development of Corti’s Organ and Auditory Systems

**DOI:** 10.3390/audiolres15040075

**Published:** 2025-06-23

**Authors:** Valeria Caragli, Valerio M. Di Pasquale Fiasca, Elisabetta Genovese, Alessandro Martini

**Affiliations:** 1 Otorhinolaryngology-Head and Neck Surgery, Audiology Program, University of Modena and Reggio Emilia, 41125 Modena, Italy; 2 Section of Otorhinolaryngology-Head and Neck Surgery, Department of Neurosciences, University of Padua, 35128 Padua, Italy; valerio.fiascadp@gmail.com; 3 Audiology Program, Department of Maternal, Child and Adult Medical and Surgical Sciences, University of Modena and Reggio Emilia, 41100 Modena, Italy; elisabetta.genovese@unimore.it; 4 Padova University Research Center “International Auditory Processing Project in Venice (I-APPROVE)”, Department of Neurosciences, University of Padua, 35128 Padua, Italy; alessandromartini@unipd.it

**Keywords:** Kölliker’s organ, function, auditory, cochlea development, ATP signalling

## Abstract

**Background**: Kölliker’s organ (KO), a transient structure in the cochlea, plays a critical role in the auditory maturation of mammals, particularly during embryonic and early postnatal development. This organ is essential for the proper differentiation and function of cochlear cells, acting as a pivotal source of signalling molecules that influence hair cell development and synaptic connectivity. **Methods**: This study systematically analyses the literature according to the PRISMA statement in order to evaluate the function roles of KO during cochlea development, reporting the molecular mechanisms and signalling pathways involved. **Results**: From our study, it emerged that KO supporting cells release adenosine triphosphate (ATP) through connexin hemichannels, initiating a cascade of intracellular calcium (Ca^2+^) signalling in adjacent inner hair cells (IHCs). This signalling promotes the release of glutamate, facilitating synaptic excitation of afferent nerve fibres and enhancing auditory neuron maturation prior to the onset of hearing. Additionally, the spontaneous electrical activity generated within KO supports the establishment of essential neural connections in the auditory pathway. The dynamic interplay between ATP release, Ca^2+^ signalling, and morphological changes in KO is crucial for cochlear compartmentalisation and fluid regulation, contributing to the formation of endolymph and perilymph. Furthermore, KO supports cellular plasticity and may provide a reservoir of precursor cells capable of trans-differentiating into hair cells under specific conditions. **Conclusions**: Dysregulation of KO function or delayed degeneration of its supporting cells has been implicated in auditory disorders, underscoring the importance of this organ in normal cochlear development and auditory function. Despite its identification over a century ago, further investigation is necessary to elucidate the molecular mechanisms underlying KO’s contributions to auditory maturation, particularly in human physiology.

## 1. Introduction

The auditory system in mammals is not fully developed at birth. Cochlear cell differentiation, the myelination process, and hearing function maturation occur over time and at different rates across species. For instance, in rats, auditory development occurs between postnatal days 12 and 14 [1], whereas in humans, it begins between the 10th and 12th weeks of gestation, and it is then largely completed by the 33rd gestational week [2]. On the contrary, the process of myelination in the human auditory system initiates around the 22nd week of gestation, continues until the 29th week, and is completed within the first year of life [3].

During this critical period of auditory development, Kölliker’s organ (KO) has been identified as playing a pivotal role.

KO is a transient structure within the cochlea, degenerating from embryonic columnar cells into cuboidal cells of the inner sulcus. KO, also referred as the greater epithelial ridge (GER), is composed of a dense array of columnar supporting cells located medial to the inner hair cells (IHCs) along the cochlear duct. It undergoes different structural transformations, aligning with the functional maturation of the cochlea and enabling the development of the mature Corti’s organ and the reception of external auditory stimuli. In various classifications, the KO or GER is further subdivided into the lesser epithelial ridge (LER) and the GER proper, based on its position and cellular characteristics. However, in many studies the terms KO and GER are used interchangeably, as they largely overlap both anatomically and functionally.

The embryological and mature cochlea organs take their names from the two scientists, Albert von Kölliker and Alfonso Corti, who first described them and who worked side by side and then published their findings a few years apart [4].

In 1863, Kölliker observed an epithelial bulge composed of a single layer of large vertical cells in calf embryos, which exhibited distinctive morphological characteristics upon closer examination. However, he was never able to determine their functional significance or ultimate fate [5]. The current knowledge on the morphological aspect of Kolliker’s organ has been analysed in a dedicated published paper [6].

Significant advancements in the understanding of KO functions have been made in recent decades. In this regard, Tritsch et al. [7,8] demonstrated that KO is responsible for the spontaneous release of adenosine triphosphate (ATP), which modulates intracellular calcium (Ca^2+^) levels in inner hair cells via intercellular gap junction protein hemichannels. This Ca^2+^-mediated mechanism triggers the release of glutamate from synaptic vesicles within inner hair cells, thereby exciting afferent nerve fibres. Through this process, KO-supporting cells transmit essential temporal and spatial information for cochlear development, facilitating synaptic refinement along the auditory pathway [9].

Recent studies revealed some of the molecular pathways involved in KO function. In this regard, it emerged that the expression of purinergic receptors, such as P2X and P2Y, is implicated in mediating ATP-dependent signalling within KO cells [10]. Furthermore, the interplay between KO and the developing tectorial membrane has been suggested as a potential regulator of cochlear hair cell maturation [11]. These interesting findings, as well as the current lack of knowledge on these topics, suggest the need for further research aimed at fully elucidating KO’s functional mechanisms. In this regard, despite its identification over a century ago, the molecular and physiological mechanisms underlying KO’s function remain inadequately understood, and its role in clinical audiology is largely unexplored. Nonetheless, given its essential role in early cochlear function, it is plausible to hypothesise that delayed degeneration or dysfunction of KO may contribute to aberrant auditory development [12], potentially resulting in congenital or acquired hearing impairments.

The objective of this study is to synthesise the existing literature on KO across mammalian species, highlighting the molecular mechanisms and the functional roles of KO for the development of mature cochlea and auditory systems.

## 2. Materials and Methods

### 2.1. Study Design

The stepwise process of systematic review was conducted using online searches on medically related databases. Before formally starting the search process, pilot searches were run on PubMED and Scopus. Preliminary searches were performed using generic search terms like “Kölliker’s organ” to ensure that relevant studies could be found in the available literature. Only English-language works were included, and limitations on publication date, settings, geographical position, and interventions of the studies were not applied. The studies were included when (1) concerning the development of Kölliker’s organ and (2) conducted on mammalian species. Inclusion and exclusion criteria are also reported in Table 1.

### 2.2. Search Methods

The analysis followed the guidelines established by the Preferred Reporting Items for Systematic Reviews and Meta-Analyses (PRISMA) 2020 Statement. Based on consultations with relevant librarians in the Pinali Medical Library of the University of Padua, search terms for Kölliker’s organ were included. The Boolean term “OR” was used to combine different ways of spelling the name of the organ (Kolliker, Kölliker’s, and Kolliker*). The last search was performed on 12 March 2024.

The most relevant and comprehensive databases for this research topic, such as PubMed, Embase, CINAHL, and Scopus, were identified (see Table 2). In selecting these databases, the research team was guided by the advice of the librarians. Using these databases in our research, all the other ones were subsequently covered. Databases were accessed through the online portal of the Pinali Medical Library. The option of searching “all search terms” was selected for each database using the search strategy outlined previously.

After performing the literature search, data were independently extracted by two authors and subsequently reviewed by a third author. Consensus on any discrepancies was achieved through discussion.

## 3. Results

**Figure 1:**
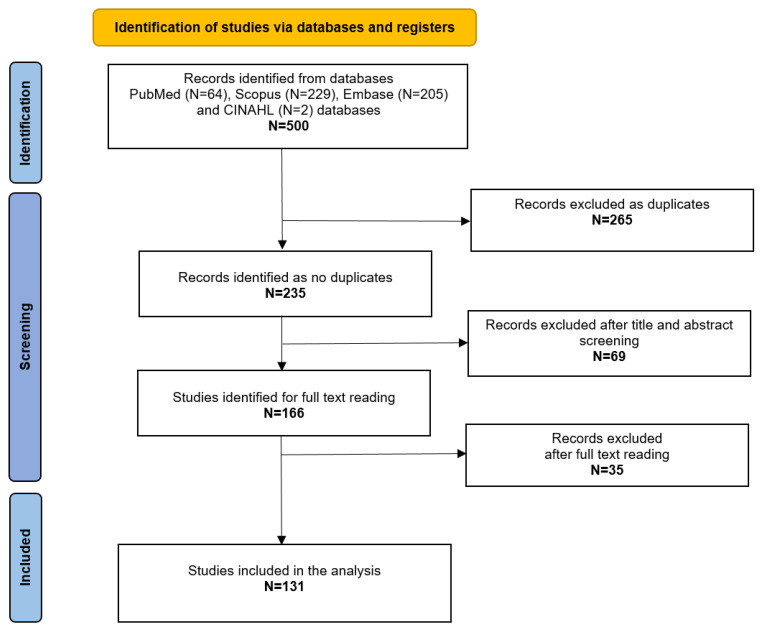
PRISMA flowchart.

A total of 500 studies were retrieved from the mentioned databases. After the exclusion of duplicates, 235 articles were evaluated through title and abstract reading, and 166 studies had their full text read by the authors. Finally, a total of 131 were included for the analysis of the study, matching the inclusion criteria [7,13,14,15,16,17,18,19,20,21,22,23,24,25,26,27,28,29,30,31,32,33,34,35,36,37,38,39,40,41,42,43,44,45,46,47,48,49,50,51,52,53,54,55,56,57,58,59,60,61,62,63,64,65,66,67,68,69,70,71,72,73,74,75,76,77,78,79,80,81,82,83,84,85,86,87,88,89,90,91,92,93,94,95,96,97,98,99,100,101,102,103,104,105,106,107,108,109,110,111,112,113,114,115,116,117,118,119,120,121,122,123,124,125,126,127,128,129,130,131,132,133,134,135,136,137,138]. The results of the screening process are reported in Figure 1.

### 3.1. Regulation of Spontaneous Cochlear Activity and Purinergic Signalling

Prior to the onset of hearing, afferent nerve fibres are active, with hair cells continuously releasing low levels of glutamate. This spontaneous activity is essential for auditory neuron survival, synaptic maturation, and auditory spatial refinement [13,14,15]. This crucial process is sustained by Kölliker’s organ (KO)-supporting cells, which contribute by generating spontaneous Ca^2+^ action potentials and in turn regulate cochlear development through a gap junction-mediated cellular network [16,17,18].

Specifically, in the second postnatal week, KO cells release adenosine triphosphate (ATP) into the endolymph via hemichannels, activating P2X and P2Y purinergic receptors on adjacent hair cells. This event triggers phospholipase C (PLC)-dependent production of diacylglycerol and inositol triphosphate (IP3), elevating intracellular Ca^2+^ and generating inward currents [7,19]. These rhythmic changes propagate radially through gap junctions, with border cells playing a critical role in initiating activity [20]. Intracellular diffusion of IP3 promotes Ca^2+^-mediated neurotransmitter release, activating spiral ganglion neurons (SGNs) and refining auditory pathways [8,21,22]. Thus, the ATP-Ca^2+^ signalling cascade sustains cochlear spontaneous electrical activity, which is essential for maturation [23].

Moreover, during cochlear maturation, the sensory epithelium differentiates into the greater epithelial ridge (GER) and the lesser epithelial ridge (LER), with Kölliker’s organ (KO) positioned medially. Inner hair cells (IHCs) arise from the GER, whereas outer hair cells (OHCs) originate from the LER [24,25].

Purinergic signalling plays a crucial role in KO cellular activity: ATP, GTP, and UTP function as neurotransmitters, gliotransmitters, and paracrine molecules, respectively [26]. P2X receptors are permeable to Na^+^, K^+^, and Ca^2+^, with subtypes P2X1-P2X7, whereas P2Y receptors are G protein-coupled receptors that activate PLC, leading to intracellular Ca^2+^ mobilisation via IP3, as mentioned before [27].

In the developing rat cochlea, P2Y receptors are expressed in both sensory and non-sensory cells, with P2Y2 and P2Y4 being crucial for GER function [10] Conversely, P2X2 and 3 receptors mediate the specific innervation of sensory cells by SGNs [10,28]. During development, type I and type II SGNs initially innervate both IHCs and OHCs, but programmed retraction occurs postnatally, guided by neurotrophins such as brain-derived neurotrophic factor (BDNF) and neurotrophin-3 [29,30]. P2X2 and 3 signalling inhibits this neurotrophic support [31]. P2X7 receptors, present from embryonic stages to adulthood, contribute to ion homeostasis and apoptosis [32], while P2X1 receptors are transiently expressed and downregulated by P10, indicating their early developmental role [33]. As a consequence, low purinergic receptor expression might occur, though ATP-induced responses persist in young KO cells [7]. Thus, neural circuit formation in the auditory system occurs before sound-driven activity, with experience-independent action potentials refining synaptic connections [8,34,35] and KO-supporting cells exhibiting synchronous spontaneous activity, potentially influencing adjacent IHCs [7].

### 3.2. Possible Mechanisms of Spontaneous Activity Generation and Mediators in Kölliker’s Organ

KO-supporting cells have a key regulator role for the spontaneous electrical and Ca^2+^ activity during auditory system development.

Recent studies have suggested two independent types of spontaneous activity in the developing auditory system: autonomous Ca^2+^ action potential generation in supporting cells and stochastic Ca^2+^ transients inducing synchronised bursts in IHCs. Specifically, IHCs generate fast Ca^2+^ transients and minibursts, while synchronised bursts are driven by intercellular supporting cell Ca^2+^ waves [36]. Spontaneous currents in early development (P0-P3) are smaller, faster, and more frequent than at later stages (P7-P10) [8].

Spontaneous activity probably involves ATP release through connexin hemichannels, particularly Cx26 and Cx30, which are abundantly expressed in KO but absent in sensory cells within Corti’s organ [37,38,39,40]. This theory is supported by the inhibitory effects of gap junction blockers, like octanol and carbenoxolone, and increased activity upon hemichannel opening in Ca^2+^-free environments [8,39,41].

Pannexins, specifically Panx1 and Panx2, also emerged as candidates for ATP release, due to their expression in the supporting cells of the adult cochlea. Although their developmental expression remains uncertain [42], it has to be mentioned that Panx1’s genetic loci are associated with DFNA11, a form of non-syndromic sensorineural deafness [43].

Periodic ATP production induces glutamate release and triggers SGNs’ action potentials [44]. Additionally, Ca^2+^ spikes persist even in Ca^2+^-free solutions, suggesting the involvement of internal stores [45]. This provides insight into KO’s spontaneous activity mechanisms.

ATP-induced activity appears to be biphasic, with P2X receptors responding faster than P2Y receptors, as demonstrated by the slower response to UTP, a P2Y agonist, compared with ATP [7]. Moreover, ATP degradation by ectonucleotidases yields adenosine, contributing to rhythmic spontaneous activity regulation [46]. ATP may also mediate gap junction coupling [47], though alternative findings suggest IHC-driven spontaneous activity independent of ATP-induced depolarisation [34]. Variations in ionic composition, such as K^+^ levels and Ca^2+^ buffering, may underlie differences between purinergic and IHC-driven theories.

Spontaneous morphological changes in KO-supporting cells, including cytoplasmic retraction and cell crenation, are tightly linked to inward currents, with 93% of such optical changes correlated to these currents [7].

Potential mechanisms for morphological changes include Ca^2+^-activated Cl^−^ channels (e.g., TMEM16A) or nonselective cation channels promoting water expulsion. Supportively, a cultured cochlea demonstrates water secretion [48]. Although evidence is lacking, contractile proteins like actin may also play a role during these events, facilitating cochlear fluid compartment (endolymph and perilymph) formation. In this process, Ryanodine receptors (RyR) regulate intracellular Ca^2+^ release, auditory transduction, spiral ganglion neuron signalling, and auditory neuron excitability [19]. Calbindin D 28K (Calb1) and S100 proteins also participate in hearing development, with Calb1 aiding intracellular calcium transport, buffering, synaptogenesis, and early Purkinje neuron activity [49,50]. Similarly, S100 proteins support neuronal plasticity [51].

Cubilin and megalin proteins are also involved, as they form an endocytotic receptor complex in early inner ear epithelia, facilitating each other’s functions. Although established ligands exist in inner ear fluids, their physiological role remains unclear [52].

Neuron-specific enolase (NSE) and calbindin (CaBP 28 kDa) exhibit specific spatiotemporal expression patterns during inner ear development [53]. NSE appears early in ganglion neurons, coinciding with afferent synapse formation at 8 weeks and displaying an apex-to-base gradient in vestibular sensory cells. Conversely, calbindin appears in vestibular ganglion neurons by 6–7 weeks and cochlear ganglion neurons by 8–9 weeks before later being restricted to cochlear hair cells (14 weeks) [49]. S100 proteins are similarly expressed, regulating neuronal plasticity [54].

Finally, Cache Domain Containing 1 (CACHD1), an α2δ-like subunit, has been recently identified as a modulator of voltage-gated calcium channels, including T-type Cav3 channels. CACHD1-deficient mice exhibit hearing and balance impairments alongside disrupted calcium homeostasis, reduced endocochlear potentials, and elevated endolymph calcium concentrations, underscoring voltage-gated calcium channels’ role in endolymph homeostasis [55].

### 3.3. Role of KO in Tectorial Membrane Formation

It has been demonstrated that human tectorial membrane (TM) fibres express collagen type II, V, IX, and XI and three unique non-collagenous glycoproteins: a-tectorin (TECTA), b-tectorin, and otogelin [56]. KO-supporting cells facilitate TM formation by producing glycoproteins and sulphated groups. During development, TM is transiently attached to KO via filamentous networks, separating at approximately P14. Thyroid hormones regulate this process by promoting KO cell survival, morphological changes, and SGN development. Deficiencies of these hormones lead to TM malformations, cochlear shortening, and improper KO separation [11,57]. Thyroxine also modulates glycoprotein synthesis, ensuring correct TM composition [58]. In hypothyroid rodents, TM thickening and glycosylation defects disrupt normal cochlear function [59,60,61].

Similarly, microtubule-associated proteins in KO undergo dynamic modifications during cochlear maturation. Although tyrosinated tubulin is initially expressed ubiquitously, it remains predominant in KO, while other modifications emerge later [62]. Developmental delays in KO degeneration, inner sulcus formation, and TM separation have been observed in congenital hypothyroidism models [63]. For instance, Pax8-deficient mice exhibit thickened TMs and stereocilia disarray, reinforcing the role of thyroid hormones in cochlear development [64].

### 3.4. Molecular Factors Influencing KO Degeneration

KO degeneration is a prerequisite for the functional maturation of Corti’s organ. Caspase-dependent apoptosis is time-dependent, with peak expression of caspase-3, caspase-8, caspase-9, and BCL family proteins in neonatal cochlear basement membranes [65,66]. Necrotic-like changes in TUNEL-positive KO cells suggest concurrent exogenous and endogenous apoptotic pathways [67]. Apoptosis and proliferation coexist during KO regression, with thyroid hormone deficiency delaying this process [58,68,69]. As mentioned before, neurotrophin receptor p75NTR is transiently expressed in KO, and it is modulated by thyroxine. Nonetheless, its role in KO apoptosis remains unclear [70,71], while the consistent role of thyroid hormones is supported again, orchestrating ordered cochlear development [72].

### 3.5. Autophagy in KO-Supporting Cells

Autophagy is a crucial process in inner ear development and cochlear maturation, as it mitigates oxidative stress and preserves hair cell morphology. KO cells exhibit distinct temporal expression patterns of autophagy- and apoptosis-related markers, also regulated by collagen family genes. Autophagy peaks at P1, preceding the bell-shaped peak of apoptotic markers (Bcl-2, caspase-3, caspase-8, and caspase-9) at P3 [73]. Organelles are degraded via autophagy before apoptosis, maintaining energy homeostasis [67].

TUNEL-positive KO cells are detected throughout the cochlear turns at P1, accompanied by decreased LC3-II, P62, and Beclin1 expression, suggesting reduced autophagic activity during KO regression. Similarly, decreased Cx26 expression impairs ATP-mediated Ca^2+^ responses, indicating a regulatory role of gap junctions in this process [74].

### 3.6. KO and Cochlear Immune Response

Macrophage recruitment into the greater epithelial ridge (GER) following programmed cell death is not essential for KO regression, as demonstrated in CX3CR1-deficient mice [75]. However, KO-supporting cells contribute to cochlear immunity by functioning as macrophage-like cells. Upon infection, virus-infected supporting cells express macrophage markers and undergo detachment, protecting adjacent hair cells. Supporting cells also phagocytose bacteria, highlighting their role in cochlear immune defence [76].

### 3.7. Molecular Signalling Pathways in Kölliker’s Organ-Supporting Cell Degeneration and Cochlear Hearing Development

The development of cochlear hearing involves multiple genes and different signalling pathways, including Sox2, Pou4f3, Atoh1, FGF, Notch, FoxG1, Strip1, mTOR, and Wnt [77,78,79,80,81,82,83,84,85,86,87,88]. However, the exact molecular mechanisms governing the degeneration of Kölliker’s organ-supporting cells remain unclear.

The ribosome and PI3K-Akt pathways regulate cell proliferation, differentiation, apoptosis, and migration [89,90,91]. The PI3K-Akt pathway is particularly implicated in cochlear hair cell regeneration [92,93]. Myc genes, which encode transcription regulators, are expressed in the inner ear and influence cellular differentiation. N-myc transcripts are notably high in the auditory nerve, Kölliker’s organ, and spiral ganglion during gestation, with roles in glial and neuronal differentiation [94,95].

The Hedgehog (HH) signalling pathway regulates cochlear sensory domain size and differentiation. The inhibition of HH signalling expands the prosensory domain, whereas Sonic Hedgehog (SHH) suppresses its formation [96].

SOX2 and pSMAD1/5/9 function as morphogenetic regulators during cochlear development. In particular, pSMAD1/5/9 establishes a stable positional framework for sensory domain specification, while SOX2 refines local differentiation patterns [97]. BMP signalling, which is mediated by Alk3/6 receptors, is essential for Corti’s organ formation and sensory-non-sensory patterning in the cochlea [98].

Somatostatin (SST) and its receptors (SSTR1 and SSTR2) are involved in cochlear maturation too. SSTRexpression peaks around P14 and declines thereafter, influencing Akt phosphorylation and hair cell survival [99].

Bmi1, a key regulator of the cell cycle and redox balance, is expressed throughout cochlear development, particularly in Kölliker’s organ and supporting cells. Its presence suggests a role in cochlea maturation and maintenance [100].

Jxc1 encodes a nuclear protein critical for Corti’s organ patterning. Mutations in Jxc1 result in supernumerary hair cells and structural duplications [101].

Gata3 is essential for prosensory domain specification and SGN survival. In this regard, tqGata3-null mutants exhibit cochlear dysmorphogenesis, increased cell death, and neuronal depletion [102].

Msi1, an RNA-binding protein, is expressed in otocyst cells and persists in supporting cells into adulthood, suggesting a role in asymmetric cell division and differentiation [103].

LaminB1 is involved in chromatin organisation and transcription, and it is localised to cochlear structures such as Kölliker’s organ and hair cells, diminishing its expression postnatally [104].

Notch signalling regulates hair cell differentiation. Notch1 and Jag1 are initially expressed in Kölliker’s organ precursor cells, while Jag2 is present in nascent hair cells. This dynamic interplay influences cochlear morphogenesis and hair cell fates [105].

### 3.8. Trans-Differentiation Potential of Kölliker’s Organ-Supporting Cells into Hair Cells

Mammalian cochlear hair cells lack a regenerative capacity in adulthood. However, Kölliker’s organ-supporting cells, which are present from the mid-embryonic to early postnatal stages, retain precursor cell properties and can trans-differentiate into hair cells [87,106,107].

#### 3.8.1. Role of Atho1 and Hes Genes

Atho1 overexpression induces KO-supporting cells to differentiate into myosin VIIa-positive hair cells, forming keratin plates and stereocilia bundles [108,109]. Specifically, Atho1 is absent postnatally in KO, while it is embryonically active from E12.5. This suggests that KO cells cease differentiation into hair cells due to Atho1 repression [110]. Conversely, Hes1 and Hes5 negatively regulate hair cell differentiation; Hes1 is expressed in KO at P0-P3 and represses Atho1, while Hes5 modulates ectopic hair cell formation [111,112].

#### 3.8.2. Atoh1 and Transcription Factor Interplay

Atoh1 overexpression induces KO-supporting cell trans-differentiation into hair cells, facilitated by the Isl1/Tub/Znf532 pathway [113,114,115]. Co-activation of Atoh1 with Pou4f3, Gfi1, Gata3, and Nymc enhances trans-differentiation, yielding mature hair cell-like phenotypes in both neonatal and mature cochleae [78,113,115].

#### 3.8.3. USP48 and Cochlear Development

USP48 is involved in DNA repair, acts as an H2A deubiquitinase, opposing BRCA1, and regulates TRAF2 stability. Thus, it influences E-cadherin-mediated junctions during cochlear development [9]. Specifically, E-cadherin expression inversely correlates with supporting cell differentiation into hair cells [116]. Thus, USP48 expression in fetal spiral ganglion and Scarpa’s neurons suggests its essential role in auditory function, as its knockdown in zebrafish results in reduced statoacoustic neurons and impaired auditory startle response [117].

#### 3.8.4. EGFR and Cochlear Cell Differentiation

EGFR plays a role in cochlear cell differentiation and response to ototoxic damage, maintaining auditory neuron homeostasis in adults [118,119]. Its absence may contribute to the cochlea’s limited regenerative capacity.

#### 3.8.5. Lgr5-Positive Progenitor Cells and Wnt Signalling

Lgr5-positive progenitors in KO generate hair cells in neonatal mouse cochlea, and they are regulated by Wnt signalling [87,106]. Lgr5 expression declines during development, persisting only in D3 cells in adults [87]. Activation of Wnt/β-catenin and inhibition of Notch pathways stimulate Lgr5 cell-mediated Myo7a-positive hair cell regeneration [120,121]. Additional regulators include Shh, Foxg1, and Hippo pathways [122,123,124,125]. However, regeneration efficiency remains low without co-activation of other pathways [93,126,127,128]. The SEC inhibitor flavopiridol reduces Lgr5 progenitor proliferation, highlighting SEC’s role in progenitor regulation. Foxg1 cKD enhances direct trans-differentiation of supporting cells into hair cells [125].

#### 3.8.6. Bmi1 and Hair Cell Survival

Bmi1 modulates neonatal cochlear-supporting cell proliferation by indirectly activating Wnt signalling and inhibiting DKK family members. It regulates redox homeostasis and ROS levels, contributing to hair cell survival [129]. Targeting Bmi1 may enhance regenerative therapies.

#### 3.8.7. Ephrin-B2 and Cell Fate Regulation

Ephrin-B2 and EphA4 regulate tissue morphogenesis in Corti’s organ, contributing to supporting cell and hair cell layer organisation [130,131]. Inhibiting Ephrin-B2 signalling allows KO-supporting cells near inner hair cells to trans-differentiate [132]. Sox2 may mediate this change [133], and Ephrin-B2 may act downstream of Notch signalling [134,135].

#### 3.8.8. Single-Cell Transcriptome Sequencing

Single-cell transcriptome sequencing confirms KO-supporting cells’ trans-differentiation potential, revealing two Cdkn1b- and Sox2-expressing precursor populations in KO’s medial and lateral regions [118,136,137,138]. These subtypes differentiate into distinct trajectories, giving rise to either outer or inner hair cells.

**Figure 2:**
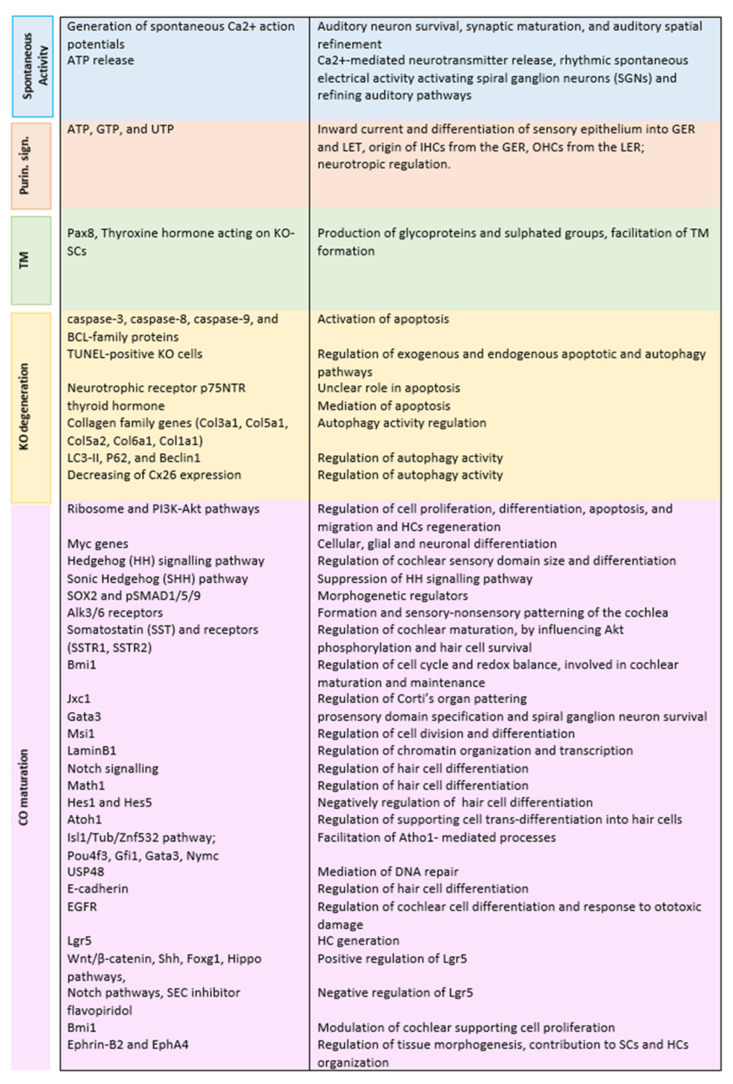
Identification of functions of KO molecular mechanisms and pathways.

Figure 2 summarises the function of the mentioned molecular mechanisms and pathways.

## 4. Discussion

This study elucidates the multifaceted role of Kölliker’s organ in the development of mammalian cochlea and auditory function. Moreover, it emphasises the contribution of KO to spontaneous cochlear activity, ATP-mediated signalling, and the potential for supporting cell trans-differentiation into hair cells. These results align with the existing literature, underscoring the significance of KO in auditory neuron survival and cochlear maturation and reinforcing the notion that spontaneous activity is crucial for refining auditory pathways [13,14,15].

The role of KO-supporting cells in generating spontaneous Ca^2+^ action potentials through a gap junction-mediated network is particularly noteworthy. This mechanism, which facilitates the rhythmic propagation of activity essential for cochlear development, parallels the findings of Tritsch et al. (2007) [7] and Nishani Dayaratne et al. (2015) [20]. Specifically, they demonstrated the impact of KO-derived ATP on hair cell activity and synaptic maturation. Similarly, the activation of P2X and P2Y receptors in adjacent hair cells shows the importance of purinergic signalling in cochlear function, a theme consistently highlighted in the literature [27,28]. Moreover, the identification of distinct types of spontaneous activity in the developing auditory system—specifically, the autonomous generation of Ca^2^^+^ action potentials in supporting cells and the stochastic Ca^2^^+^ transients in IHCs—brings new insights into the interplay between these cell types. Eckrich et al. (2018) [36] corroborated the idea of intercellular calcium waves in supporting cells driving synchronised activity in IHCs, further emphasising the intricate signalling dynamics that govern cochlear development.

The proposed mechanisms of ATP release through connexin hemichannels and the involvement of pannexins align with previous studies, indicating the role of these channels in cochlear function [39,42]. The biphasic response of P2 receptors to ATP and the subsequent degradation to adenosine suggest a tightly regulated signalling environment, consistent with the findings of Vlajkovic et al. (1998) [46] regarding ATP’s role in spontaneous activity regulation.

The hypothesised involvement of morphological changes in KO-supporting cells, including cytoplasmic retraction and cell crenation, in relation to inward currents offers a novel perspective on the cellular adaptations occurring during cochlear maturation. These observations resonate with the work of Tritsch [7], who noted the correlation between spontaneous activity and morphological changes in supporting cells. The contribution of Ca^2^^+^-activated Cl^−^ channels and contractile proteins in mediating these alterations remains to be fully elucidated, but existing studies suggest that these pathways may play a role in fluid compartment formation in the cochlea [48].

The interplay of hormonal regulation, particularly thyroid hormones, in the developmental processes of tectorial membrane formation and KO degeneration is supported by a wealth of research [11,57,58].

Furthermore, the temporal expression patterns of markers associated with autophagy and apoptosis provide insight into the balance between cell survival and degeneration within KO. The observation that autophagy peaks prior to apoptotic marker expression aligns with the findings of Liu [67], suggesting a protective role for autophagy in cochlear development.

Interestingly, the immune response of KO-supporting cells, described as macrophage-like, adds another layer of complexity to their functional repertoire. This observation parallels recent reports highlighting the involvement of supporting cells in cochlear immunity, particularly in response to infection [76].

Finally, the potential for KO-supporting cells to trans-differentiate into hair cells underscores their regenerative capabilities, a topic of great interest in cochlear biology. The regulatory roles ofAtho1, Hes genes, and various signalling pathways, including Wnt and Notch, provide a framework for understanding the molecular mechanisms underlying this trans-differentiation [87,106,107,111]. The identification of specific progenitor populations within KO-supporting cells, as evidenced by single-cell transcriptome sequencing, further substantiates the notion that KO retains regenerative potential throughout development [136,137].

Our findings contribute to a comprehensive understanding of the roles played by Kölliker’s organ in cochlear development, spontaneous activity regulation, and potential regenerative processes. These insights not only reinforce the existing literature but also pave the way for future research aimed at harnessing the regenerative potential of KO-supporting cells for therapeutic applications in hearing loss.

## 5. Future Directions

Future research on Kölliker’s organ and its role in auditory development and function is essential, and it should focus on several critical areas to enhance our understanding of and the therapeutic potential in hearing loss. First, the molecular mechanisms governing the transition from KO to the mature organ of Corti need further elucidation. Studies should aim to identify the specific signalling pathways and transcription factors involved in KO degeneration and their interactions with surrounding cellular environments. This could involve advanced imaging techniques and single-cell RNA sequencing to capture dynamic changes at the cellular and molecular level during cochlear maturation.

Given the potential for KO-supporting cells to trans-differentiate into hair cells, future investigations should explore gene editing techniques to manipulate key regulators such as Atoh1, aiming to enhance hair cell regeneration in vivo. The role of epigenetic modifications in this trans-differentiation process also warrants exploration, as it may provide insights into the regulation of progenitor cell plasticity.

Research should also investigate the influence of environmental factors, such as acoustic stimulation during critical developmental windows, on the functional characteristics of KO and its supporting cells. Understanding how these external stimuli interact with KO’s intrinsic signalling pathways could reveal new strategies for auditory rehabilitation.

Moreover, the immune functions of KO-supporting cells present an exciting avenue for exploration. Investigating their role in cochlear responses to injury, inflammation, and infection could lead to novel therapeutic approaches for sensorineural hearing loss.

Finally, the integration of computational models and system biology approaches could facilitate a holistic understanding of the KO’s dynamics within the cochlear ecosystem, potentially leading to predictive models for auditory development and the impact of therapeutic interventions. By addressing these areas, future research may significantly advance our understanding of KO and its implications for congenital and acquired hearing impairments, paving the way for innovative treatments and regenerative strategies in audiology.

## 6. Conclusions

Kölliker’s organ plays a pivotal role in cochlear maturation and auditory function. The existing literature highlights its significance as a transient structure that undergoes substantial remodelling during embryonic and early postnatal stages, thus facilitating crucial communication between supporting cells and hair cells.

Through intercellular signalling mechanisms based on ATP release and calcium signalling, Kölliker’s organ generates spontaneous activity within its cells, which is essential for neural circuit formation in the auditory system and for the trans-differentiation of supporting cells into hair cells. Furthermore, the interplay of genetic factors, signalling pathways, and environmental influences, such as thyroid hormones, reveals a complex regulatory network governing cochlear development.

Despite its identification over a century ago and the promising avenues it offers for regenerative medicine, the molecular mechanisms underlying the structure and function of Kölliker’s organ remain inadequately understood, particularly in humans. Therefore, in light of KO’s great potential as a key player in cochlear development and auditory function, future research aimed at elucidating its intricate biology is essential. Understanding its regenerative capabilities is also crucial for proposing novel therapeutic strategies aimed at restoring hearing. The integration of advanced technologies, such as single-cell transcriptomics and gene editing, could succeed in exactly explaining Kölliker’s organ’s functions and its contributions to auditory health and hearing restoration.

## Figures and Tables

**Table 1:** Inclusion and exclusion criteria.

	Criteria
Inclusion	-Studies analysing Kölliker’s organ -Human or mammalian subjects
Exclusion	-Non-English papers

**Table 2:** Search queries for literature review.

Database	Query
Pubmed	“Kolliker’s organ” [All Fields] OR “Kolliker organ” [All Fields]
Scopus	TITLE-ABS-KEY(“Kolliker’s organ” OR “Kolliker organ”)
Embase	“Kolliker’s organ” OR “Kolliker organ”
CINAHL	“Kolliker’s organ” OR “Kolliker organ”

## Data Availability

No new data were created or analysed in this study. Data sharing is not applicable to this article.

## Abbreviations

The following abbreviations are used in this manuscript:

KO	Kölliker’s organ
PLC	Phospholipase C
IP3	Inositol triphosphate
SNG	Spiral ganglion neuron
GER	Greater epithelial ridge
LER	Lesser epithelial ridge
IHC	Inner hair cell
OHC	Outer hair cell
TM	Tectorial membrane
